# LncRNA-SERB promotes vasculogenic mimicry (VM) formation and tumor metastasis in renal cell carcinoma

**DOI:** 10.1016/j.jbc.2024.107297

**Published:** 2024-04-18

**Authors:** Shuai Tang, Fangmin Chen, Jianghui Zhang, Fan Chang, Zheng Lv, Kai Li, Song Li, Yixi Hu, Shuyuan Yeh

**Affiliations:** 1 College of Medicine, Nankai University, Tianjin, China; 2 Department of Urology, Nankai University Affinity The Third Central Hospital, Tianjin, China; 3 Department of Urology, The Third Central Hospital of Tianjin, Tianjin, China; 4 Departments of Urology, Pathology, and The Wilmot Cancer Institute, University of Rochester Medical Center, Rochester, New York, USA; 5 The Sex Hormone Research Center and Department of Urology, China Medical University/Hospital, Taichung, Taiwan

**Keywords:** noncoding RNA, ERβ, TKI, vasculogenic mimicry, clear cell renal cell carcinoma, metastasis

## Abstract

A growing body of evidence shows that vasculogenic mimicry (VM) is closely related to the invasion and metastasis of many tumor cells. Although the estrogen receptor (ER) can promote initiation and progression of renal cell carcinoma (RCC), how the downstream biomolecules are involved, and the detailed mechanisms of how ER expression is elevated in RCC remain to be further elucidated. Here, we discovered that long noncoding RNA (LncRNA)-SERB is highly expressed in tumor cells of RCC patients. We used multiple RCC cells and an *in vivo* mouse model for our study, and results indicated that LncRNA-SERB could boost RCC VM formation and cell invasion *in vitro* and *in vivo*. Although a previous report showed that ERβ can affect the VM formation in RCC, it is unclear which factor could upregulate ERβ. This is the first study to show LncRNA-SERB can be the upstream regulator of ERβ to control RCC progression. Mechanistically, LncRNA-SERB may increase ERβ *via* binding to the promoter area, and ERβ functions through transcriptional regulation of zinc finger E-box binding homeobox 1 (ZEB1) to regulate VM formation. These results suggest that LncRNA-SERB promotes RCC cell VM formation and invasion by upregulating the ERβ/ZEB1 axis and that therapeutic targeting of this newly identified pathway may better inhibit RCC progression.

Renal cell carcinoma (RCC) accounts for approximately 2% to 3% of adult malignant tumors and currently ranks ninth (1), accounting for 90% of all renal malignancies (2). Worldwide, there are about 209,000 new RCC cases per year, and clear cell renal cell carcinoma (ccRCC) is the most common type, accounting for approximately 80% of all kidney cancers.

The 5-year survival rate of metastatic ccRCC is <20%, which highlights the need for further biologic and therapeutic studies of this disease (3, 4). The current treatment for metastatic RCC mainly consists of antiangiogenic therapies/tyrosine kinase inhibitors, such as treatment with sunitinib and pazopanib, as well as vascular endothelial growth factor receptor which have been shown to be effective with curative effects (5). However, majority of patients eventually acquire resistance and relapse, and some patients even have inherent resistance to targeted therapy (6). Despite the relatively high rates of response to combinations of PD-1 and/or CTLA-4 axis inhibition in RCC that form the new standard-of-care, most patients with RCC do not receive durable clinical benefit from these therapies (7). Hence, a comprehensive understanding of the mechanisms of RCC progression and angiogenesis is required to develop better therapeutic strategies.

Vasculogenic mimicry, referred to as VM, is closely related to clinical stage, pathological grade, and tumor invasion and metastasis (8, 9) and plays a role similar to vascular function (10). Distinct from classical tumor angiogenesis, VM provides an alternative channel of blood supply for tumor cells independent of endothelial cells (11). As reported, epithelial-mesenchymal transition (EMT) and cancer stem cells are potential mechanisms contributing to VM formation (12), and some EMT-associated transcription factors, including Snail1, Nodal, zinc finger E-box binding homeobox 1 (ZEB1), ZEB2, and Twist1, may also be involved in VM formation (11, 13, 14). VM formation does not involve vascular endothelial cells but can form lumen-like structures in the cellular microenvironment *via* cell deformation and protruding pseudopodia. This kind of microtubule-like structure can accommodate the passage of individual red blood cells, providing continuous nutrition or energy support for the tumor at the microenvironmental level (15). Thus, the increased VM phenomenon may lead to the failure of antitumor vascular endothelial therapy.

Gender differences in the incidences of cancers have been found in almost all human cancers (16), suggesting a possible important role for sex hormone receptors in regulating tumor growth. It has been reported that the estrogen receptors (ERs) are involved in many important physiological functions, as well as tumor development. Two classes of ERs exist: ERα and ERβ. ERβ, also known as ESR2, is considered to be more extensively expressed in ccRCC than ERα (17). Previously, ERβ was recognized as a prognostic factor in breast cancer (18), and plays a protective role in a variety of tumors, such as lung cancer, colon cancer, and breast carcinoma (19, 20, 21, 22). Interestingly, in other types of tumors, it has been reported that ERβ promotes the progression of tumors, including prostate cancer and RCC (1, 17, 23, 24, 25). These results suggest that the ERβ could play differential roles in different types of tumors, and even in the same tumor, the functions of ERβ may be altered at different stages. In this study, we aimed to elucidate whether the formation of VMs in RCC is related to the activation of ERβ, and to identify the signaling pathway associated with the ERβ-regulated VM formation.

Among all noncoding RNA (ncRNA) families, long ncRNAs (LncRNAs) have a length greater than 200 nucleotides. Since the discovery of the first LncRNA, a body of knowledge has demonstrated pivotal roles of LncRNAs in regulating gene expression (26). The transcripts produced by 4% to 9% of the mammalian genome sequence are LncRNAs, while the corresponding protein-coding RNA ratio is 1% (27). In the past decade, it has been demonstrated that the dysregulated LncRNA profile is widely involved in the pathogenesis of many diseases, including cancer, metabolic disorders, and cardiovascular diseases (28). In particular, many LncRNAs have been revealed to play an important role in tumor growth and metastasis, including lung, breast, liver, and colorectal cancers (29, 30, 31), as well as hematological malignancies, and neuroblastoma (32, 33). The latest research also found that certain LncRNA may have important roles in the drug resistance of RCC (34). Although LncRNAs are of great significance in participating in a variety of tumors, and related research is progressing rapidly, the function of most LncRNAs and how they affect VM formation and tumor progression in RCC remain to be further investigated.

Here, we demonstrate that the LncRNA-SERB can promote ERβ expression by binding to promoter region of LncRNA-SERB and that a high level of ERβ can lead to the formation of VM through transcriptional regulation of ZEB1. Thus, the formation of this LncRNA-SERB/ERβ/ZEB1 signaling axis may contribute to tumor metastasis and disease progression.

## Results

### Higher LncRNA-SERB is associated with ERβ expression and can predict the stage and survival of ccRCC patients

Previous reports have shown that ERβ, but not ERα, could play important roles in RCC progression (1, 25, 35). The results of the UALCAN database showed that patients with higher ESR2 expression had worse survival rates for kidney renal clear cell carcinoma (KIRC) and were independent of sex (Fig. S1*A*). Even within the same tumor grade, patients with higher ESR2 expression had a worse prognosis for survival (Fig. S1*B*). To further clarify the factors that affect the pathogenesis of KIRC (RCC) patients and affect the ERβ expression, we used interaction prediction software (http://rtools.cbrc.jp/) to analyze the differentially expressed LncRNAs in cancer and normal tissues in The Cancer Genome Atlas (TCGA) database (Table. S1) and screened out candidates that may affect their downstream ERβ/ESR2.

**Figure 1:**
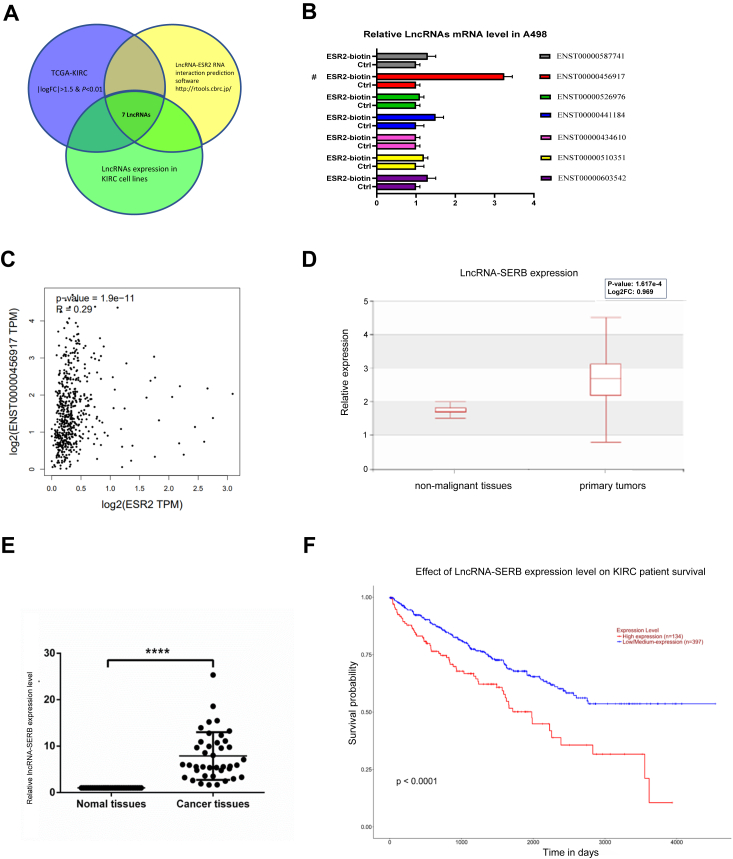
**Higher LncRNA-SERB is associated with ERβ expression and can predict the stage and survival of ccRCC patients.***A*, by merging TCGA database and candidates that may regulate ESR2 using interaction prediction software, 7 LncRNAs candidates met the requirements and were screened out. *B*, LncRNA-SERB was selectively pulled-down by the biotinylated-ERβ/ESR2 through RNA immunoprecipitation assay among those candidates. *C*, the correlation analysis of database shows a predicted correlation between ERβ and LncRNA-SERB (R = 0.29). *D*, Human Cancer Database results show that the expression of LncRNA-SERB in primary RCC tumors is higher than that in normal tissues. *E*, clinical sample test results show that the mRNA level of LncRNA-SERB in cancer tissues is higher than that in para-cancerous normal tissues from our collections. *F*, UALCAN database show that patients with higher LncRNA-SERB expression levels also have a worse survival prognosis. ERβ, estrogen receptor β; LncRNA, long noncoding RNA; RCC, renal cell carcinoma; TCGA, The Cancer Genome Atlas.

The results showed that the expression of 2682 LncRNAs in cancer and noncancerous tissues is significantly different, and 15 of them may be directly bound to the ERβ/ESR2 promoter (Table. S2) However, due to the low expression, eight of the LncRNAs could not be detected in renal tumor cells (Fig. S2), so only seven candidates met the requirements (Fig. 1*A*). Among those seven candidates, we found that only the LncRNA with specific ER β binding, (LncRNA-SERB/ENST00000456917) could be pulled down by the biotin of anti-sense mRNA of ERβ/ESR2 through RNA pull-down analysis (Fig. 1*B*). Previously, we analyzed the database GEPIA 2 (http://gepia2.cancer-pku.cn) to predict the correlation between ERβ and LncRNA-SERB using Spearman correlation coefficient. The results further corroborated that there is a positive correlation between the expressions of ERβ and LncRNA-SERB, although the correlation (R) is weak (Fig. 1*C*). This result supported our conjecture that the expression of ERβ/ESR2 may be regulated by LncRNA-SERB, based on later analyses. Through interaction prediction software analysis results, LncRNA-SERB/ENST00000456917 may influence ERβ *via* targeting its 3′UTR (Fig. S3). By querying Human Cancer Metastasis Database (https://hcmdb.i-sanger.com/) for the expression of LncRNAs in tissue microarrays of all types of KIRC patients including those with distant metastases and nondistant metastases, we found that the expression of LncRNA-SERB in primary tumors is higher than that in nonmalignant tissues (Fig. 1*D*).

Next, 40 clinical samples were collected to evaluate the database-analyzed result, and we also found that the expression of LncRNA-SERB in cancer tissues was significantly higher than that in adjacent noncancerous specimens (Fig. 1*E*). Data from the UALCAN database (http://ualcan.path.uab.edu/analysis.html) show that patients with higher LncRNA-SERB expression levels also have a worse survival prognosis (Fig. 1*F*).

In preexperiments, we confirmed that the regulation of ERβ expression can affect the formation of VM and invasion in RCC (Fig. S4). Results from Figure 1, *A*–*F* and Figs S1–S3, indicate that there is a direct RNA-RNA interaction and positive correlation between ERβ and LncRNA-SERB in ccRCC. LncRNA-SERB has a higher expression in renal tumors, and the higher expression of LncRNA-SERB predicts a worse prognosis for ccRCC patients.

### LncRNA-SERB upregulates invasion and VM formation in ccRCC cells

**Figure 2:**
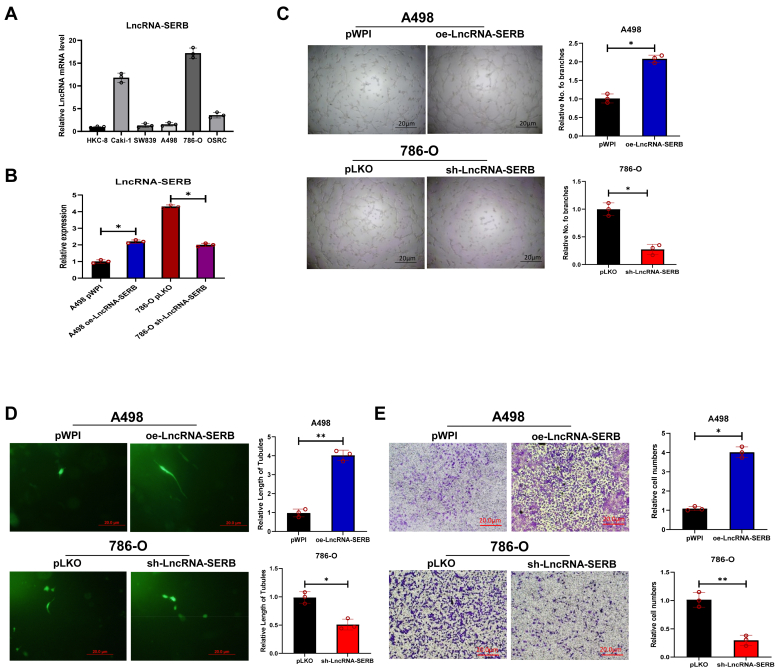
**LncRNA-SERB promotes the malignancy of ccRCC cells *via* upregulating invasion and VM formation.***A*, the qRT-PCR was performed to detect the expression of LncRNA-SERB in different cell lines. *B*, the qRT-PCR results show that LncRNA-SERB was successfully overexpressed (oe) in A498 cells transduced by lentiviral LncRNA-SERB, and LncRNA-SERB was knocked down (sh) in 786-O cells transduced by lentiviral shRNA of LncRNA-SERB. *C*, the 2D Matrigel-based tube formation assay proved that VM formation increased when LncRNA-SERB was overexpressed in A498 cells. Conversely, when this LncRNA was knocked down in 786-0 cells, the formation of VM is significantly reduced. *D*, 3D VM formation results indicate when LncRNA-SERB was overexpressed in A498 cells, the cell length of tubules increased, while knocking down this LncRNA in 786-O cells has the opposite result. *E*, invasion assays proved that high expression of LncRNA-SERB leads to increased cell invasion, and vice versa. For (*C*–*E*), quantitation is on the *right*. Three independent assays were performed in triplicate in the above data. Data are presented as mean ± SD. ∗*p* < 0.1, ∗∗*p* < 0.01. ccRCC, clear cell renal cell carcinoma; LncRNA, long noncoding RNA; qRT-PCR, quantitative real-time PCR; VM, vasculogenic mimicry.

To study the effect of LncRNA-SERB on tumor cells cytology, we tested its expression levels in different ccRCC cells relative to normal proximal tubule epithelial cells. The results showed that the expression level of LncRNA-SERB was the highest in 786-O cells, while lower in A498 and SW839 cells (Fig. 2*A*). Comparing TCGA databases, we found that LncRNA-SERB expression is higher as patients' clinical stage or tumor grade increased (Fig. S5*A*). To study the role of LncRNA-SERB, we selected two cell lines, A498 and 786-O, as experimental subjects. This is because these two cell lines not only represent the low and high expression of LncRNA-SERB, but also the gene ESR2, which is closely related to LncRNA-SERB, also shows low and high expression in these two cell lines (Fig. S6*A*). We ectopically increased the expression (oe) of this LncRNA in A498 cells and knocked down (sh) this LncRNA in 786-O cells. The result showed that LncRNA-SERB level increased by 1.1 times after overexpression, while the expression level decreased by 1.3 times after knocking down LncRNA-SERB (Fig. 2*B*). The 2D Matrigel-based tube formation assay proved that VM formation increased when LncRNA-SERB was overexpressed in A498 cells. Conversely, when this LncRNA was knocked down in 786-O cells, the formation of VM was significantly reduced (Fig. 2*C*). In addition, we validated the results using 3D VM formation assay. Consistently, the cell length of tubules increased when overexpressing LncRNA-SERB in A498 cells, and the cell length of tubules reduced while knocking down of LncRNA-SERB in 786-O cells (Fig. 2*D*). To further illustrate the influence of LncRNA-SERB on cell metastasis, the transwell Matrigel invasion assays were performed. Invasion assays show that high expression of this factor leads to increased cell invasion (Fig. 2*E*).

Together, the data from Figure 2, *A*–*E* suggest that LncRNA-SERB has a relatively high expression in tumor cells, and this factor plays an important role in promoting VM formation, collagen I based 3D VM formation, and invasion.

### ERβ can reverse the effect of LncRNA-SERB on VM formation

**Figure 3:**
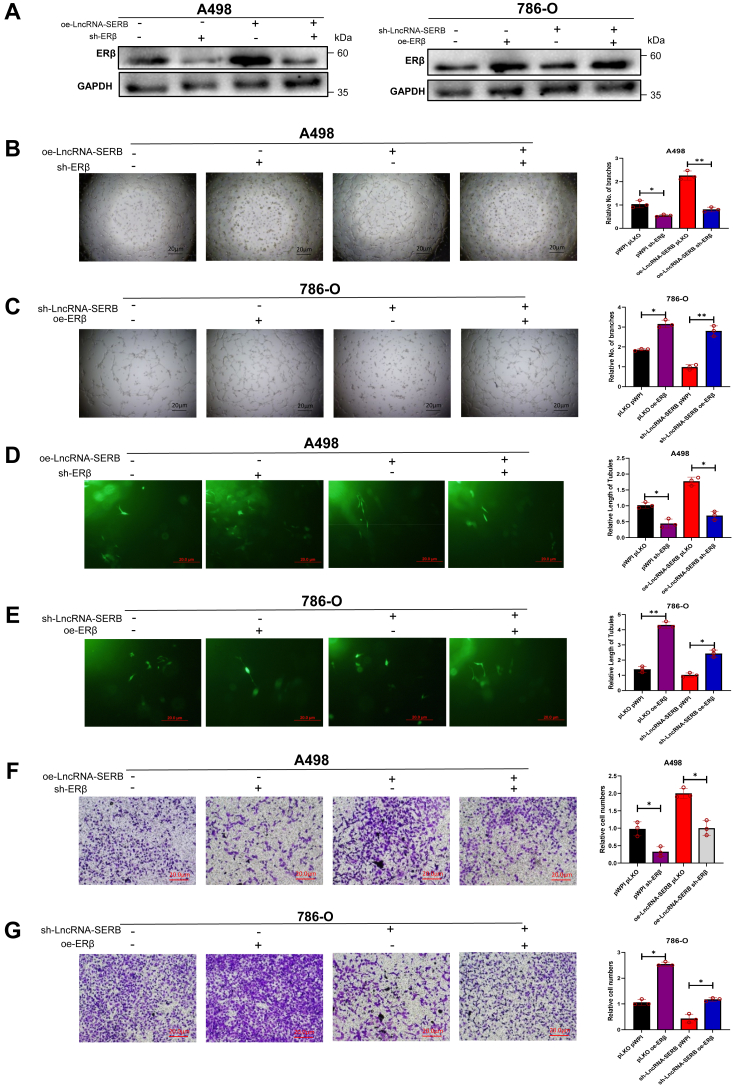
**ERβ is the down-stream effector and modulation of ERβ can control the LncRNA-SERB promoted- VM formation.***A*, overexpressing LncRNA-SERB and knocking down ERβ at the same time in A498 cells (*left panel*). Knocking down LncRNA-SERB and overexpressing ERβ in the 786-O cell line (*right panel*). *B*, overexpressed LncRNA-SERB can increase VM formation, while knocking down ERβ can effectively reverse the effect. *C*, knockdown of LncRNA-SERB in 786-O cells can reduce VM formation, while upregulation of ERβ can reverse this effect. *D*, results of 3D collagen I-induced tube formation assay show that overexpressed LncRNA-SERB can increase cell length of tubules, while knocking down ERβ can reverse it in A498 cells. *E*, in 786-O cells, sh-LncRNA-SERB decreases cell length of tubules, while oe-ERβ can rescue it. *F*, matrigel invasion assay show that the increased invasion ability promoted by oe-LncRNA-SERB in A498 cells can be reversed by knockdown of ERβ. *G*, the decreased invasion ability *via* knockdown of LncRNA-SERB (by sh-LncRNA-SERB) in 786-O can be rescued by oe-ERβ. For (*B*–*G*), quantitation is on the *right*. Three independent assays were performed in triplicate in the above data. Data are presented as mean ± SD. ∗*p* <0.1, ∗∗*p* < 0.01. ERβ, estrogen receptor β; LncRNA, long noncoding RNA; VM, vasculogenic mimicry.

Both ERβ and LncRNA-SERB have relatively low expression in A498 cells and relatively high expression in 786-O cells (Figs. 2*A* and S6*A*), and LncRNA-SERB can regulate ERβ expression both in RNA level and protein level (Fig. S6, *B*–*D*). To evaluate that ERβ is an important downstream effector of LncRNA-SERB, we next performed the functional rescue experiment. We chose A498 cell line to overexpress LncRNA-SERB, then with or without knockdown of ERβ investigated whether ERβ can reverse the effect of LncRNA-SERB (Fig. 3*A*, left). Similarly, we knocked down LncRNA-SERB and overexpressed ERβ in the 786-O cell line (Fig. 3*A*, right). The VM formation experiments results showed that overexpressed LncRNA-SERB can increase VM formation, while knocking down ERβ can effectively reverse the LncRNA-SERB increased VM formation (Fig. 3*B*). Knockdown of LncRNA-SERB in 786-O cells can reduce VM formation, while increased ERβ can reverse this effect (Fig. 3*C*). Similarly, the results of 3D collagen-tube formation assay, referred to as 3D VM formation assay, also showed that the ability of LncRNA-SERB to enhance the cell length of tubules can be reduced by shERβ and the sh-LncRNA-SERB inhibited tubular length is reversed by overexpression of ERβ (Fig. 3, *D* and *E*). Furthermore, the upregulated and downregulated LncRNA-SERB modulated invasion can be reversed by shERβ or oeERβ, respectively (Fig. 3, *F* and *G*). These results reflect the ability of LncRNA-SERB/ERβ signals in regulating tumor metastasis to distant places (36).

Together, results from Figure 3, *A*–*G* suggest that ERβ can reverse the effects of LncRNA-SERB regulated 2D and 3D VM formation, and invasion of ccRCC, indicating that ERβ is a downstream effector of LncRNA-SERB.

### Mechanism dissection of how ERβ can alter VM formation: *via* modulating ZEB1 expression

**Figure 4:**
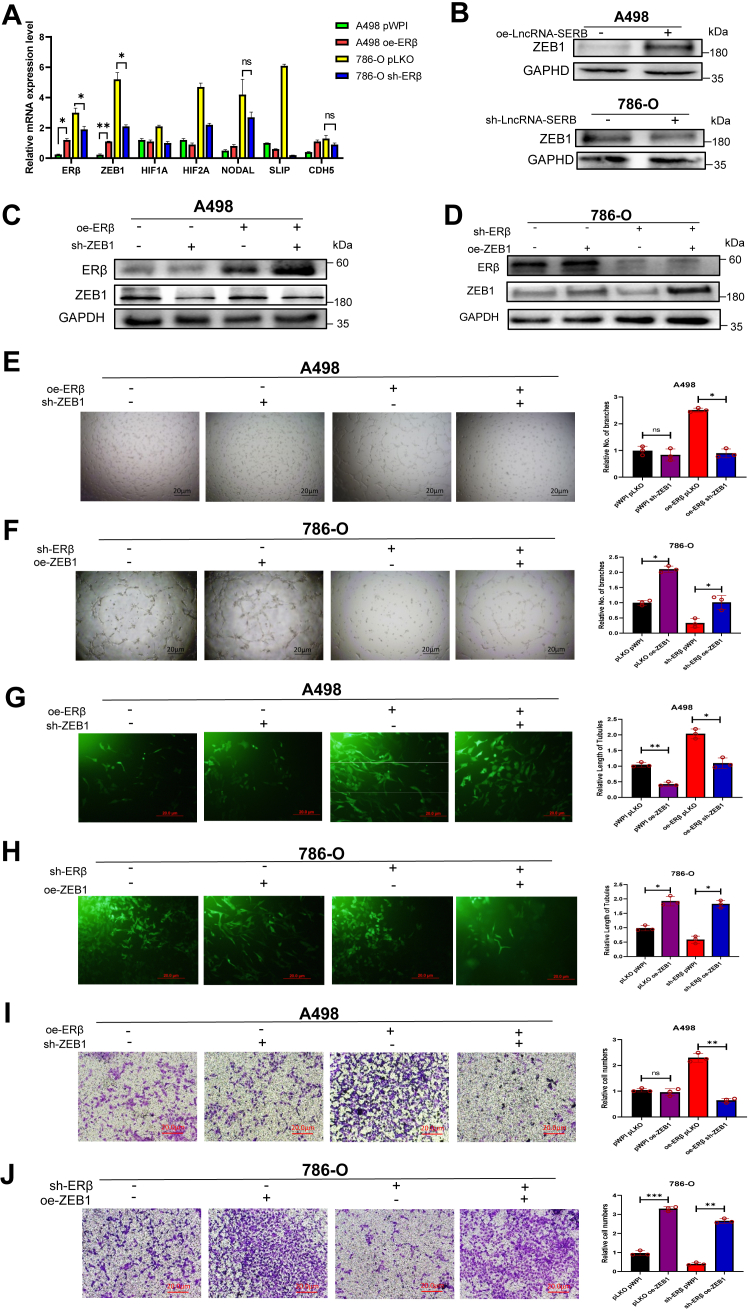
**Mechanism dissection of how LncRNA-SERB/ERβ can alter VM formation: *via* modulating ZEB1 expression.***A*, at the mRNA level, ZEB1 is specifically related to ERβ compared to the other six VM marker genes, which suggests that ERβ is likely to affect the VM formation in RCC through regulating ZEB1. *B*, at the protein level, ZEB1 was indeed increased after overexpression of LncRNA-SERB in A498 cells. In contrast, after knocking down LncRNA-SERB in 786-O cells, results show that the expression of ZEB1 decreased. *C*, Western blot assays were performed on A498 cells transduced with pWPI+pLKO, pWPI+shZEB1, oeERβ+pLKO, and oeERβ+shZEB1. *D*, Western blot assays on 786-O cells transduced with pLKO+pWPI, pLKO+oeZEB1, shERβ+pWPI, and shERβ+oeZEB1. *E*, in A498 cells, VM formation was significantly increased when ERβ was overexpressed, but this process could be effectively reversed by sh-ZEB1. *F*, in 786-O cells, when ERβ is knocked down, VM formation is reduced, but overexpression of ZEB1 can restore this reduction effect. *G* and *H*, in A498 cells, 3D collagen I-induced tube formation results show that ZEB1 can effectively reverse the ERβ-promoted cell length of tubules. *I* and *J*, in the invasion assay, we have seen similar results to VM formation, then still ZEB1 can rescue the effect of shERβ on the invasiveness of A498 (*I*) and 786-O cells (*J*). RCC cells. For (*E*–*J*), quantitation is on the *right*. Three independent assays were performed in triplicate in the above data. Data are presented as mean ± SD. ∗*p* < 0.1, ∗∗*p* < 0.01, ∗∗∗ *p* < 0.001. ERβ, estrogen receptor β; LncRNA, long noncoding RNA; RCC, renal cell carcinoma; VM, vasculogenic mimicry; ZEB, zinc finger E-box binding homeobox 1.

To further study how ERβ specifically affects VM formation, we have found 6 VM marker genes to study their relationship with ERβ (37, 38). By regulating the expression of ERβ in different ccRCC cell lines, we found that the change of ZEB1 is directly related to ERβ (Fig. 4*A*), which indicates that ERβ is likely to affect the VM formation in RCC through ZEB1. The genes, other than ZEB1, either their expressions were not associated with ERβ expression, or changes of their expressions were not statistically significant. In order to confirm the existence of the LncRNA-SERB/ERβ/ZEB1 signal axis, we performed assays on A498 cells and showed that ZEB1 was indeed increased after LncRNA-SERB overexpression. In contrast, after knocking down LncRNA-SERB in 786-O cells, results show that the expression of ZEB1 decreased (Fig. 4*B*).

To further confirm ERβ can induce VM formation *via* altering the ZEB1 expression, we applied rescue experiments with adding ZEB1-shRNA (shZEB1) and ZEB1- complementary DNA (cDNA) (oeZEB1) into A498 and 786-O cells, respectively (Fig. 4, *C* and *D*). The 2D Matrigel-based VM tube formation assay results indicate VM formation was significantly increased when ERβ was overexpressed, but this process could be reversed by shZEB1 (Fig. 4*E*). As ERβ and ZEB1 expressions are relatively low in A498 cells, knockdown of ZEB1 did not show any significant difference in VM formation compared with the control group in A498 cells (Fig. 4*E*). In 786-O cells with high endogenous ERβ expression, VM formation is reduced when ERβ is knocked down, and ectopic expression of ZEB1 can effectively restore the shERβ-mediated VM reduction in 786-O cells (Fig. 4*F*). The following 3D collagen 1-induced tube formation assay data also showed that ZEB1 can effectively reverse the effect of ERβ on the cell length of tubules (Fig. 4, *G* and *H*). In the invasion assay, we have seen similar results that ZEB1 can rescue/reverse the shERβ-reduced invasiveness of RCC cells (Fig. 4, *I* and *J*). Clinical data from TCGA also indicate that ZEB1 expression is correspondingly higher in patients with higher clinical stages or tumor grades, which is consistent with our experimental results (Fig. S5*B*).

Together, results from Figure 4, *A*–*J* suggest that ERβ may function *via* modulating ZEB1 expression to promote the Matrigel-coated 2D and collagen I-based 3D VM formation and invasion in RCC cells.

### ERβ upregulates the ZEB1 expression *via* transcriptional regulation

**Figure 5:**
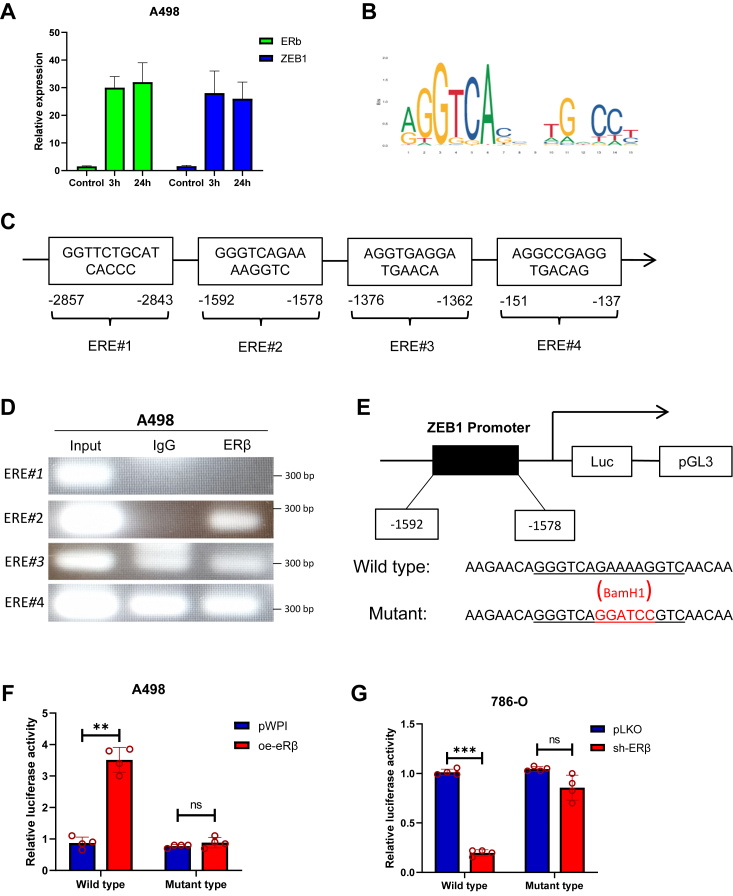
**Mechanism dissection of how ERβ alters ZEB1 expression: *via* transcriptional regulation.***A*, the expression levels of ERβ and ZEB1, after A498 cells were treated with 10 nM E2, were significantly higher than control group, and there was no significant difference in the levels of the two genes whether they were treated with E2 for 3 h or 24 h. *B*, putative EREs motif locations on ZEB1 promoter and sequences are shown. *C*, there are four putative EREs located within the ZEB1 promoter region (ERE*#1* at −2857nt to −2843nt, ERE*#2* at −1592nt to −1578nt, ERE*#3* at −1376nt to −1362nt, and ERE*#4* at −151nt to −137nt). *D*, chromatin immunoprecipitation (ChIP) assay results revealed that ERβ could bind to the ERE*#2* located at the −1592nt to −1578nt bp from the transcriptional start site of ZEB1. *E*, the core sequence of ERE*#2* was mutated by replacing it with the BamH1 restriction site. Next, the promoter with ERE#2 WT or with the ERE#2 mutant ZEB1 was cloned into the pGL3 luciferase reporter plasmid. *F*, luciferase reporter assay results revealed that overexpressing ERβ in the A498 cells led to increasing the luciferase reporter activity in WT, but not mutant type. *G*, knocking down the ERβ can significantly reduce the luciferase activity in 786-O cells transfected with the WT reporter gene, but there is no statistical difference in the cells with the mutated reporter gene. Four independent analyses were performed in the above data. Data are presented as mean ± SD. ∗∗*p* < 0.01, ∗∗∗ *p* < 0.001, ns = not significant. ERβ, estrogen receptor β; ERE, estrogen response element; ZEB, zinc finger E-box binding homeobox 1.

Although regulating the expression of ERβ can affect the changes of ZEB1 mRNA levels, it is still unclear whether ZEB1 is directly regulated by ERβ. Our hypothesis is that if ERβ regulates ZEB1 step by step through other factors, signal transmission will be relatively slow, while transcriptional regulation will respond quickly. We first hypothesize that ERβ may regulate ZEB1 *via* promoter regulation. To test this possibility, we compared the ZEB1 mRNA expression levels after treating A498 cells cultured in charcoal-stripped fetal bovine serum media with E2 for 3 h and 24 h. The results showed that the expression levels of ERβ and ZEB1 after treated with E2 were significantly higher than control group, and there was no significant difference for cells with 3 h or 24 h of treatment (Fig. 5*A*). This result leads us to speculate that ERβ may regulate ZEB1 at the transcriptional level. We next applied the Ensembl websites to search for the estrogen response elements (EREs) in the 3 kb region of ZEB1 promoter *via* JASPAR database analysis (Fig. 5*B*). There are four putative EREs located within the ZEB1 promoter region (ERE*#1* at −2857nt to −2843nt, ERE*#2* at −1592nt to −1578nt, ERE*#3* at −1376nt to −1362nt, and ERE*#4* at −151nt to −137nt) (Fig. 5*C*). We then performed the chromatin immunoprecipitation (ChIP) assay and results revealed that ERβ could selectively bind to the ERE*#2* located at the −1592 to −1578 bp from the transcriptional start site of ZEB1. ERE*#3 and ERE#4* are also detected in immunoglobulin G (IgG) pull-down complex, suggesting that the binding of ERE#3 and ERE#4 are nonspecific (Fig. 5*D*). Furthermore, we mutated this key sequence of ERE*#2* and replaced it with the BamH1 restriction site, then inserted the WT promoter and the mutant promoter region of ZEB1 into the pGL3 luciferase plasmid (Fig. 5*E*) sequences are shown in Fig. S7, *A*–*C*. As we expected, luciferase reporter assay results revealed that overexpressing ERβ in the A498 cells led to increasing the luciferase reporter activity in WT, but not the ERE-mutated promoter (Fig. 5*F*). In contrast, knocking down the ERβ can significantly reduce the luciferase activity in 786-O cells transfected with the WT reporter gene, but there is no statistical difference in the cells with the mutated reporter gene (Fig. 5*G*).

Together, the results of Figure 5, *A*–*G* and Fig. S7, *A*–*C* indicate that ERβ increases the expression of ZEB1 through transcriptional regulation and directly binds to ERE#2 (−1592nt to −1578nt) on the 5′ promoter of ZEB1.

### Preclinical study using *in vivo* mouse model to study the role of LncRNA-SERB/ERβ/ZEB1 axis in ccRCC VM formation

The orthotopic ccRCC xenograft mouse model was used to prove the above *in vitro* cell line data using the *in vivo* preclinical mouse RCC model. We generated luciferase stably transfected A498 cells. Next, we overexpressed cells with or without LncRNA-SERB and with or without ICI 182,780 or mock control with ten mice/group for four groups as follows: group 1: oe- LncRNA-SERB + mock; group 2: oe-LncRNA-SERB + ICI 182,780; group 3: pWPI + mock and group 4: pWPI+ ICI 182,780.

**Figure 6:**
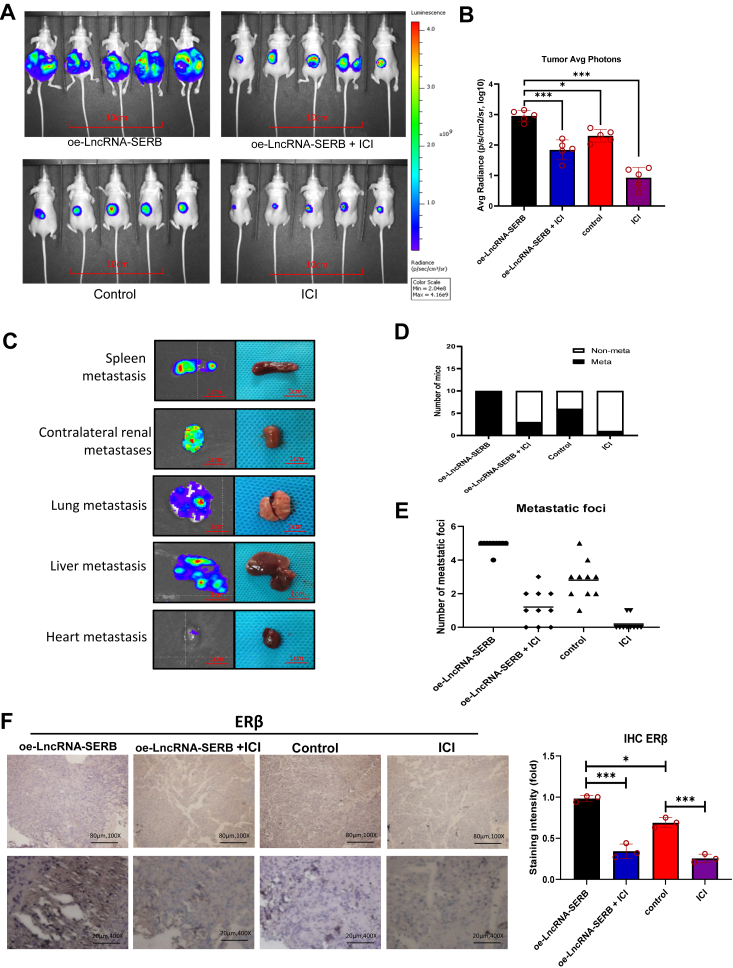
**Preclinical study using *in vivo* mouse model to study the role of LncRNA-SERB/ERβ/ZEB1 axis in ccRCC VM formation.***A*, representative IVIS imaging of female nude mice inoculated with 1 × 10^6^ A498-Luc cells into the left kidney capsule (n = 10/group) and then treated with: (1) oe-LncRNA-SERB; (2) oe-LncRNA-SERB + ICI 182,780; (3) pWPI; and (4) ICI 182,780. Tumor progressions were evaluated *via* IVIS. Five representative mouse IVIS bioluminescent images are shown. *B*, tumor average photons for ccRCC from xenograft mice described above. *C*, representative organ bioluminescent images showing metastasis to spleen, right kidney, lung, liver, and heart metastasis. *D*, quantification of the metastases in the four groups of mice. *E*, quantification of the total metastatic foci for each group. *F*, representative images of IHC staining for ERβ in mice tumors. The scale bar represents 80 μm, 100× and 20 μm, 400×. For (*F*), quantitation is on the right. Data are presented as mean ± SD. ∗*p* < 0.1, ∗∗∗ *p* < 0.001. ccRCC, clear cell renal cell carcinoma; ERβ, estrogen receptor β; IHC, immunohistochemical; IVIS, *in vivo* imaging system; LncRNA, long noncoding RNA; VM, vasculogenic mimicry; ZEB, zinc finger E-box binding homeobox 1.

A total of 1 × 10^6^ A498-Luc cells ± oe-LncRNA-SERB were inoculated into the left kidney capsule of nude mice and 1-week later groups 1 and 3 treated with mock and groups 2 and 4 treated with ICI 182,780. Tumor progressions were evaluated *via in vivo* imaging system for 3 weeks. Results showed a dramatic increase of metastatic luciferase signal in the oe-LncRNA-SERB+mock group. Importantly, treating with ICI 182,780 can partly block/reverse the oe-LncRNA-SERB-promoted ccRCC metastasis (Fig. 6*A*). The results revealed that the ICI 182,780 can partly block/reverse the oe-LncRNA-SERB-increased ccRCC growth (Fig. 6*B*). Consistently, the results of metastasis foci (as seen in the spleen, right kidney, lung, liver, and heart) also showed a similar conclusion that LncRNA-SERB increased the ccRCC metastasis (Fig. 6*C*) and the inclusion of sh-ERβ can partly block/reverse the oe- LncRNA-SERB -increased ccRCC metastasis, including the number of metastasis foci (Fig. 6, *D* and *E*). Immunohistochemical staining of these ccRCC xenograft tumors also showed that oe-LncRNA-SERB led to an increase in intracellular ERβ, while mice treated with ICI significantly reduced tumor volume and the number of metastases and correspondingly lower levels of ERβ (Fig. 6*F*).

**Figure 7:**
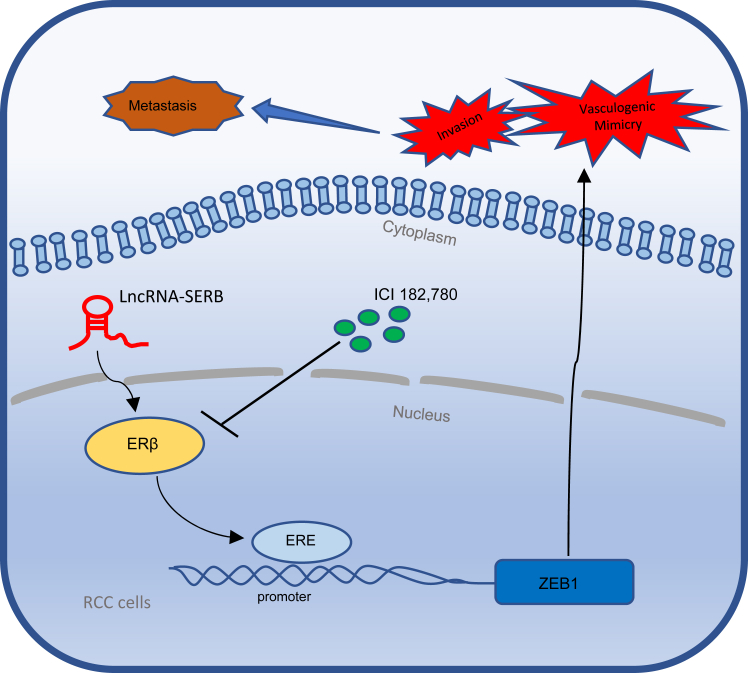
**Targeting LncRNA-SERB/ERβ/ZEB1 axis could prevent VM formation in the tumor tissues and consequently suppress tumor progression and metastasis.** The cartoon illustrates the LncRNA-SERB can enhance the expression of intracellular ZEB1 through upregulating ERβ. Activation of LncRNA-SERB/ESR2/ZEB1 signaling axis will directly lead to promoting VM formation in the tumor, and ultimately enhance the invasiveness and distant metastasis of RCC. The antagonist of ERβ, ICI 182,780, may benefit patient survival by inhibiting the expression of ERβ and thus blocking the transmission of this signaling axis. ERβ, estrogen receptor β; LncRNA, long noncoding RNA; RCC, renal cell carcinoma; VM, vasculogenic mimicry; ZEB, zinc finger E-box binding homeobox.

Together, results from our preclinical study using the *in vivo* mouse model (Fig. 6, *A*–*F*) agree with the above *in vitro* cell lines studies and demonstrated that targeting ERβ could suppress ccRCC metastasis *via* regulation of the LncRNA-SERB/ERβ/ZEB1 signals (Fig. 7).

## Discussion

The concept of VM, epithelial cell-independent angiogenesis, has received increasing attention "since it was first reported by Maniotis in 1999" (15). Epithelial-dependent angiogenesis has long been thought to be the only blood replenishment triggered by proangiogenic factors. The discovery of VM reinforces the theory of tumor neovascularization, and in 2017 Velez first observed the formation of VM using the 3D collagen I induced tube formation assay method (39). Targeting a key factor regulating VM formation in tumors may be a novel antiangiogenic therapy that blocks tumor cell progression.

ERβ has been found to play important roles in the progression of many hormone-related cancers, including breast, prostate, bladder, ccRCC, and so on (17, 40, 41, 42). Notably, ERβ may play different roles in different types of tumors. For example, highly selective ERβ agonists can attenuate the viability of breast cancer cell lines *in vitro* (43), and ERβ activation has also been observed in prostate cancer to inhibit tumor cell proliferation and suppress MYC proto-oncogene transcription (44). However, it has a clear cancer-promoting effect in hepatocellular carcinoma (45). Even in the related studies of renal cancer, there are quite different views on the role of ERβ. Related research by Yu *et al.* (46) showed that estrogen can inhibit the progression of renal cell cancer cells, but an increasing number of other studies have found that the role of ERβ in promoting the progression of renal cancer is more significant (25). In this study, we further confirmed that ERβ can promote the invasion and metastasis of ccRCC, whether *in vivo* or *in vitro* experiments. This conclusion is also consistent with the results of the clinical data from the TCGA database. Not only that, but we also found that Vimentin protein is also elevated with ERβ (Fig. S5, *A* and *B*), and Vimentin upregulation reflects the high differentiation plasticity of tumor cells (47).

In addition to clarifying the specific role of ERβ in renal cancer, we would like to know what causes the increase of ERβ in ccRCC, which is of great significance for elucidating the pathogenesis of the disease and targeted therapy. We turned our attention to LncRNAs, which could play important roles to regulate gene transcription and modification (48). A total of 8293 LncRNAs were included in the study by comparing the LncRNA sequencing results of cancer and para-cancerous samples in the TCGA database, but only 2682 had significant expression differences. Not all of these noncoding RNAs can regulate ERβ expression. After interactive prediction analysis and screening, seven LncRNAs were finally identified that may be involved in regulating the expression of ERβ. Fortunately, the results of the LncRNA/mRNA pull-down assay showed that LncRNA-SERB-ENST00000456917 could bind to the 3′UTR of the ERβ promoter and increase its expression (Fig. 2*B*). The LncRNA-SERB (ENST00000456917), also named as MIR155 host gene (MIR155HG), which can be transcriptionally activated by promoter insertion at a common retroviral integration site in B-cell lymphomas (49). To confirm the elevated expression of LncRNA-SERB in ccRCC, we collected clinical samples from 40 ccRCC patients after surgery. The results showed that the expression of LncRNA-SERB in cancer tissue was significantly higher than the normal level (Fig. 2*E*). We performed phenotypic validation through *in vitro* VM formation and invasion assays, experiments as well as clinical data showed that LncRNA-SERB and ERβ can significantly promote ccRCC VM formation.

Although we have now established that ERβ can lead to the formation of VM, as we mentioned before, it is expressed in many tumors and plays different roles, which suggests that it may not directly contribute to VM formation, our next objective was to find its downstream gene targets in RCC. We set our sights on oncogenes that may be directly involved in the regulation of VM formation. For this reason, we reviewed large number of oncogenes that may be related to EMT, which is pivotal in malignant tumor progression and VM formation (50). Through the mutual verification of our previous experiments and literature reports, six oncogenes closely related to VM were finally identified (51, 52, 53, 54). Interestingly, when we regulate the expression of ERβ in cells, only ZEB1 is highly consistent with the former trend, which gives us reason to consider whether ZEB1 is the downstream target gene of ERβ in regulating VM formation in RCC (Fig. 4*A*). ZEB1 is a key factor for cell fate determination, tumor initiation, cancer cell plasticity, and metastatic dissemination (55). In our study, we found that ZEB1 could reverse ERβ-enhanced ccRCC VM formation and invasion. Mechanism studies reveal that ERβ can promote ZEB1 expression both in RNA and protein level indicating ERβ may play a role in directly regulating its downstream ZEB1 through transcriptional regulation (Fig. 5*D*). Further ChIP assays confirmed that ERβ could bind to the promoter of ZEB1 for posttranscriptional regulation (Fig. 5, *F* and *G*).

Significantly, we find that ERβ plays an important role in VM formation and invasion that is the signal of metastasis and poor prognosis. When we exogenously added E2, both VM formation and invasion were significantly increased, but when we added the estrogen inhibitor ICI 182,780, this phenomenon can be inhibited. Subsequent *in vivo* experiments also confirmed that the increase of LncRNA-SERB can promote the expression of ERβ and lead to tumor invasion and distant metastasis. When we used ER-specific inhibitors, the key molecule ERβ could be effectively blocked, tumor growth and diffusion were partially reversed also (Fig. 6, *A*–*E*). This shows that ERβ may be a potential therapeutic target. In addition, ZEB1 can effectively reverse the role of ERβ as a switch in regulating VM, suggesting that it is more likely to be a key factor in the prognosis of RCC, although more relevant studies are needed to confirm this. Over the past few years, emerging evidence has implicated ZEB1 in drug resistance in multiple cancers (56, 57, 58), but it is unclear whether ZEB1 induced drug resistance is related to its direct promotion of VM formation.

In summary, LncRNA-SERB could promote ccRCC VM formation and invasion *via* altering the LncRNA-SER/ERβ/ZEB1 signals and targeting these newly identified signals may help physicians to develop a new therapy to better suppress the ccRCC progression.

## Experimental procedures

### Patients and samples

A total of 40 tumor and para-cancerous specimens from patients with a pathologic diagnosis of ccRCC were collected from the Department of Urology, the Third Central Hospital of Tianjin affiliated to Nankai University (Tianjin, China) between October 2017 and July 2020. All patients were admitted to the hospital for surgery. After signing the “Scientific Ethical Consent”, tumor and para-cancer samples were collected during the operation and immediately frozen at −80 °C. Our research was approved in advance by the Hospital Institutional Review Board.

### Cell culture and reagents

The A498, 786-O, SW839, Caki-1, OSRC-2, HKC-8, and HEK293T cell lines were purchased from the American Type Culture Collection (ATCC). After 3 to 4 cycles of growth the cells were frozen in liquid nitrogen tanks in at least three batches, and the use period after resuscitation did not exceed 4 weeks. According to ATCC’s protocol, all cell lines used in the article have been authenticated as *mycoplasma* and bacteria-free and periodically reauthenticated by PCR. Cells were cultured in Dulbecco's modified Eagle's medium (DMEM) media with 1% penicillin (25 units/ml) and L-glutamine, containing 10% fetal bovine serum. All cells were maintained in a humidified 5% (v/v) CO_2_ incubator at 37 °C.

### Lentivirus packaging and cell transfection

The cDNA was cloned into the Pac1 or Pme1 site of the pWPI lentiviral vector, and shRNA was cloned into the Age I and EcoRI sites of the pLKO.1 lentiviral vector. The HEK293T packaging cells were transiently transfected with pMD2G, psPAX2 and pWPI vector/pWPI-cDNA, or pLKO vector/pLKO-shRNA to produce lentiviral particles. The supernatants containing lentiviral particles were collected 48 h posttransfection of HEK293T cells. The lentiviral supernatants were then filtered with 0.45 μm filters and used to infect RCC cells for 48 h.

### 2D Matrigel-based tube formation assay

We placed Matrigel Matrix (BD Biosciences) at 4 °C overnight in advance, and evenly added 50 μl to each well of a 96-well plate for incubation at 37 °C for 2 h. Subsequently, 100 μl of cells were resuspended in serum-free DMEM and loaded onto the surface of Matrigel at 3 × 10^4^ cells/well. After incubation at 37 °C for 4 h, the formation of tubule-like structure was analyzed using a microscope (Olympus) by determining the length of the tubules in each field of view, counting the average length of 3 to 5 random fields of view in each well. The renal tubules were analyzed by ImageJ software (https://imagej.net/software/imagej) to quantitatively measure the number of branches of formed VM tubes (59).

### 3D collagen I-induced tube formation assay

Cells suspended in DMEM media were first mixed with 10× reconstitution buffer, 1:1 (v/v), and then with soluble rat tail type I collagen (Corning) to a final concentration of 6 mg/ml. NaOH (1 M) was used to neutralize the pH (pH 7, 10–20 μl 1 μM NaOH) (39). Then 200 μl of the mixture was loaded into a 48-well culture plate and incubated in a 5% (v/v) humidified CO_2_ incubator at 37 °C for 2 h. Next, 3 × 10^4^ cells (in 500 μl of DMEM media) were added to the surface of the collagen mixture. After 7 days of culture, we measured the tubule lengths.

### Cell invasion assay

The transwell assay was used to determine the invasive ability of RCC cells. Approximately 1 × 10^5^ cells were resuspended in serum-free media and inoculated in the upper chambers precoated with Matrigel (BD Corning) for 24 h. The invaded cells were fixed with 4% paraformaldehyde and stained with 1% toluidine blue. The invaded cells were counted in different fields of each tested well (100×). All experiments were carried out with three replicates each time, and each experiment was repeated at least three times, independently. Data represent the mean ± SD of three replicate experiments.

### RNA extraction and quantitative real-time PCR (qRT-PCR) analysis

Total RNAs were isolated using TRIzol reagent (Invitrogen). Superscript III transcriptase (Invitrogen) was used for reverse transcription of 2 μg of total RNA. We used the Bio-Rad CFX96 system and SYBR green to perform quantitative real-time PCR to determine the mRNA expression levels of the target genes. The expression level was normalized to the GAPDH level by using the 2-ΔΔCt method. The relative sequences of the primers are listed in Table S3*A*.

### Western blot

The indicated cells were washed twice with cold PBS, and then cell lysis buffer was added to lyse the cells, The same amount of protein (30–50 μg) from each sample was mixed with protein loading dye, boiled, and separated on a 10 to 12% SDS/PAGE gel, and then transferred to polyvinylidene fluoride (PVDF) membranes (Millipore). The PVDF membrane was blocked with 5% skim milk for 1 h, and then incubated with the specific primary antibody at 4 °C overnight. The following primary antibodies were used at a 1:1000 dilution; GAPDH (Santa Cruz, #sc-166574), ERβ (R&D Systems, #PPZ0506), TCF8/ZEB1 (CST, #3396), and Vimentin (CST, #5741). The next day, anti-rabbit or anti-mouse IgG secondary antibody was used at a concentration of 1:5000 for 2 h at room temperature. The PVDF membrane was then rinsed with 1× Tris Buffered Saline with Tween for 5 min (3 times). The protein signals (on the membrane) were visualized using an enhanced chemiluminescence detection system (Thermo Fisher Scientific).

### LncRNA/mRNA pull-down assay

The cells were collected by centrifugation, washed with cold 1× PBS, and then lysed with 1 ml cell lysis buffer with 100 units RNase inhibitor (M0307S, NEB). The cell lysates were frozen at −80 °C for 30 min, thawed on ice, and centrifuged to collect the supernatant. After being evenly divided into two groups, 500 PM biotin-labeled anti-sense oligonucleotide targeting ERβ/ESR2 (5′-TCT GTC TCC GCA CAA GGC GGT ACC C-3′) was added to the experimental group, and the same amount of 1× Tris-EDTA (TE) buffer was used as a control group and rotated at 4 °C overnight. This mixture was incubated with 30 μl of streptavidin agarose beads by rotation for 2 h at 4 °C, centrifuged, and then washed with RNA immunoprecipitation buffer ten times. Total RNA was extracted according to the RT-PCR method, and then quantitative PCR analysis was performed to detect the levels of RNAs of interest as indicated. Sequences of the anti-sense ERβ/ESR2 biotin probe and screened candidate RNA primers are listed in Table S3*B*.

### Luciferase reporter assay

An approximately 3kb promoter of ZEB1 (ENST00000361642) was cloned into the PGL3 basic vector (Promega). By mutating the key site of the ERβ/ESR2 binding site in the ZEB1 5′-promoter to the BamHI cleavage site (GGA TCC), we constructed a mutagenic promoter PGL3 vector. A498 and 786-O cells were seeded in 24-well plates, and cDNA was transfected with Lipofectamine 3000 transfection reagent (Invitrogen) according to the manufacturer's instructions. Thirty-six to forty-eight hours after transfection, luciferase activity was measured using a dual luciferase assay (Promega) according to the manufacturer's manual. pRL-TK was used as an internal control. Data are expressed as the mean ± SD. Experiments were performed in triplicate and at least three repetitions.

### Chromatin immunoprecipitation assay

In brief, A498 cells were cross-linked with 3% formaldehyde and lysed in lysis buffer. Then, we sonicated the samples with a predetermined power to yield genomic DNA fragments with lengths of 300 to 1000 bp. Protein A/G beads were preincubated to remove nonspecific binding. The experimental group and the control group were treated with anti-ERβ antibody (2.0 μg) or IgG control, respectively, and placed at 4 °C with overnight rotation. Specific primer sets designed to amplify a target sequence within the human ZEB1 promoter are listed in Fig. S7, *A*–*C*. PCR products were analyzed by agarose gel electrophoresis.

### *In vivo* mouse RCC studies

Forty 5-week-old athymic BALB/c-nu female mice were divided into four groups for injection with luciferase-labeled cells under the renal capsule. Two groups (group 1 and group 2) of mice were injected with LncRNA-SERB/A498-Luc cells, and two groups (group 3 and group 4) were injected with Vec/A498-Luc cells as a control. After 1 week of tumor growth, groups 1 and 3 were treated with 10 μl of 1× 10-2 M ICI 182,780 mixed with 90 μl of sesame oil, and groups 2 and 4 were treated with 100 μl of sesame oil as a mock control and injected intraperitoneally into each mouse every other day for 2 weeks. The noninvasive *in vivo* imaging system was performed once a week. Then, the mice were killed, and the tumors were excised and photographed. All experimental procedures were conducted under the guidance and approval of the Experimental Animal Ethics Committee of Tianjin Third Central Hospital affiliated with Nankai University.

### Immunohistochemical staining

Tumor tissues were fixed in 10% formalin, embedded in paraffin, cut into 5 μm sections, deparaffinized in a 65 °C incubator for 30 min, and then deparaffinized in xylene and hydrated in ethanol. For antigen retrieval, tissue slides were then placed in sodium citrate (pH 6) and microwaved for 10 min. Then 3% H_2_O_2_-methanol was added to tissues to inactivate endogenous peroxidase. Sections were then incubated with ERβ antibody (Novus Biologicals, 14C8) in the same blocking solution at a dilution concentration of 1/300 for 45 min at room temperature. Diaminobenzidine chromogen showed immunoreactivity, and slides were counterstained in hematoxylin, dehydrated, cleared, and permanently mounted with resin mounting medium (Thermo Fisher Scientific) (60).

### Database using

The TCGA database was used to search for noncoding RNAs associated with tumor heterogeneity. The UALCAN database (http://ualcan.path.uab.edu/analysis.html) is an easy-to-use interactive portal for in-depth analysis of TCGA gene expression data that can help identify candidate biomarkers for specific cancer subclasses with diagnostic, prognostic, or therapeutic significance. UALCAN database is mainly used for query genetic relationship with the prognosis of patients. We also evaluated the correlation between ESR2 and LncRNA-SERB using GEPIA 2 online analysis software (http://gepia2.cancer-pku.cn). Interaction prediction software (http://rtools.cbrc.jp/cgi-bin/RNARNA/index.pl) was used to predict the binding site between LncRNA-SERB and ESR2. Ensembl database (https://asia.ensembl.org/index.html) is recognized as the global biological data resources and ELIXIR core data resources; this tool can be used to predict gene regulatory regions.

### Statistical analysis

Unless otherwise stated, all results are expressed as the mean ± SD, and the experiment was independently repeated at least 3 times with data points in triplicate. Statistical significance was determined by SPSS 22 software (IBM Corp, www.ibm.com/support/pages/spss-statistics-220-available-download) and GraphPad Prism 8.0 (GraphPad Software, Inc, www.graphpad.com/updates/prism-802-release-notes) using Student's *t* test. Differences were considered statistically significant when the *p* value was less than 0.05 (∗*p* < 0.05, ∗∗*p* < 0.01, ∗∗∗*p* < 0.001).

## Conclusions

In the present study, we explore the underlying mechanisms, and our new findings show that LncRNA-SERB could promote the expression of ERβ *via* targeting its 3′UTR in ccRCC and increased ERβ could promote VM formation *via* binding to the promoter of ZEB1. Next, we used preclinical mouse tumor model to prove that adding the Food and Drug Administration-approved anti-estrogen ICI 182,780 can effectively reduce tumor volume and inhibit metastasis. In this study, we demonstrate that LncRNA-SERB could promote RCC VM formation and invasion *via* altering the LncRNA-SERB/ERβ/ZEB1 signals and targeting these newly identified signals may help physicians to develop a new therapy to better suppress the RCC progression.

## Data availability

The data used and analyzed in this study are available from the corresponding author upon reasonable request.

### Institutional Review Board statement

Ethical Approval and consent to participate Ethical consent was approved by the Human Subjects Research Ethics Review Committee of the Tianjin Third Central Hospital affiliated to Nankai University. Written informed consent was obtained from each patient prior to sample collection. Animal experiments were approved by the Animal Experiment Ethics Committee of Nankai University.

## Supporting information

This article contains supporting information.

## Conflict of interest

The authors declare that they have no conflicts of interest with the contents of this article.
