# Supplementary material for: LncRNA-SERB promotes vasculogenic mimicry (VM) formation and tumor metastasis in renal cell carcinoma
Source: J Biol Chem. 2024 Apr 18;300(5):107297. doi: 10.1016/j.jbc.2024.107297 (PMC11126803; doi:10.1016/j.jbc.2024.107297)
Supplement: Supporting Figures S1–S7 [file mmc1.pdf]

A

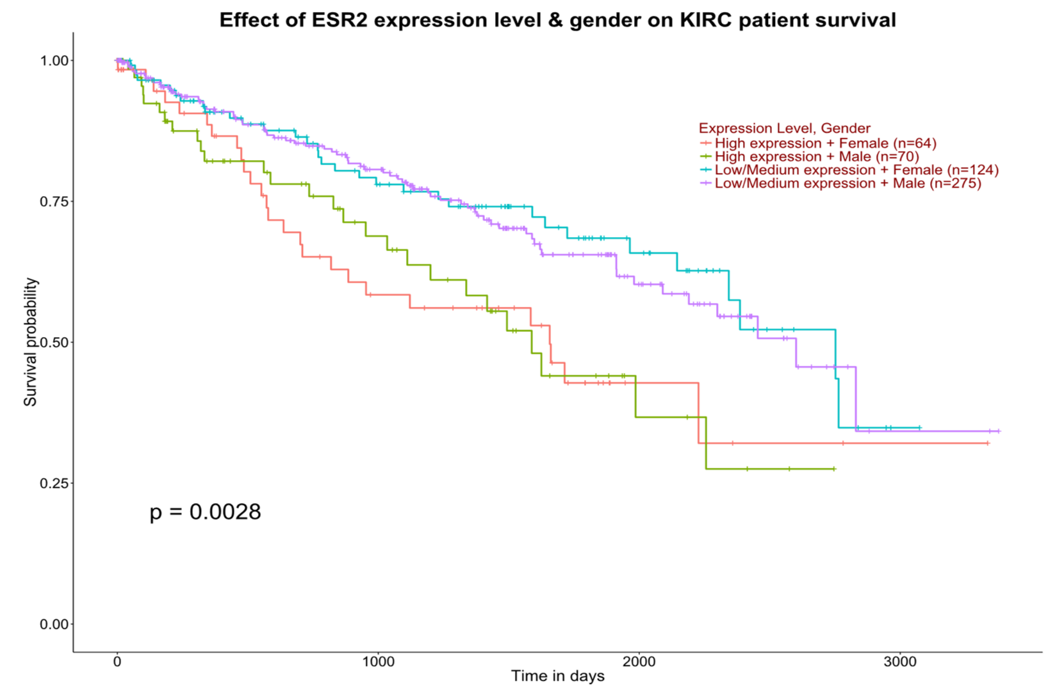

B

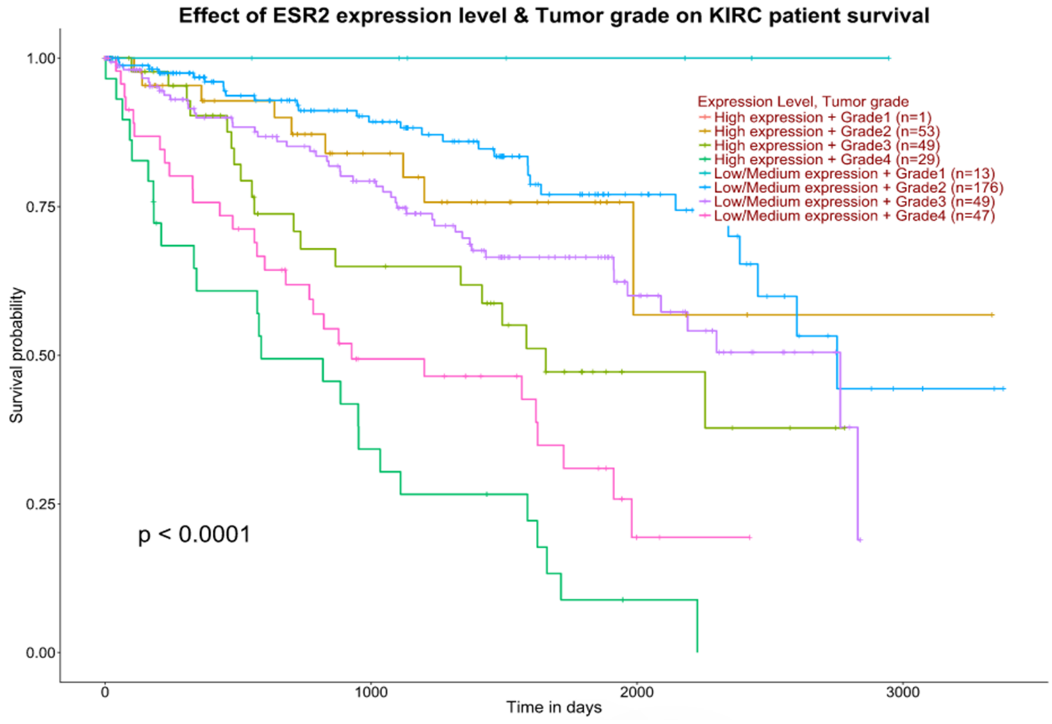

Supplementary Fig. S1. Effect of ESR2 expression on KIRC patient survival

(A) Effects of different ERβ (ESR2) expression levels and gender on survival in the UALCAN database (*p*=0.0028).
(B) Effect of ESR2 expression on survival rate in patients with different tumor grade, higher ESR2 levels in patients with the same pathologic stage predict a worse prognosis (*p* < 0.0001).

Tang et al., Supplementary Fig. S2

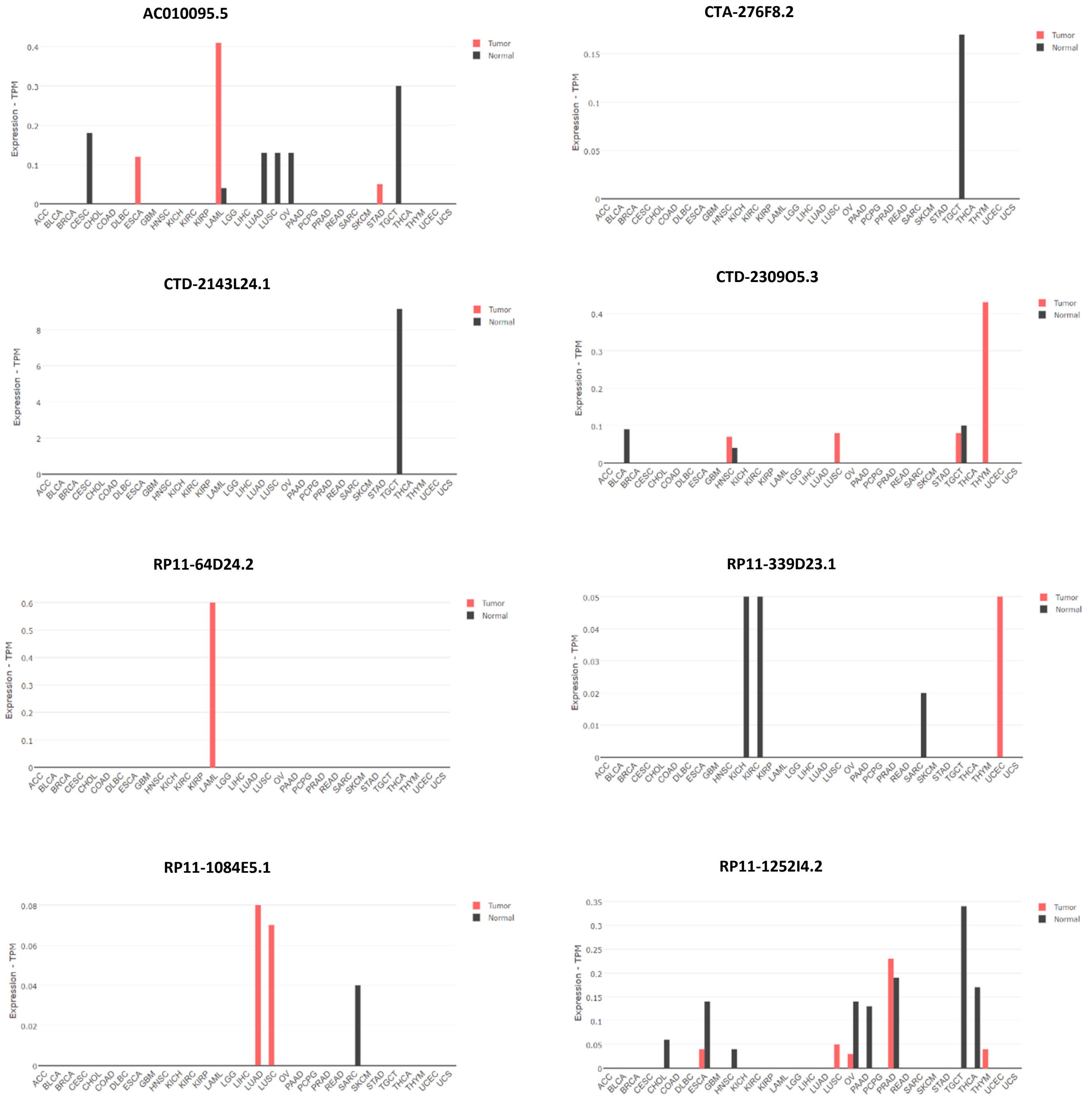

Supplementary Fig. S2. Eight LncRNAs were undetectable in renal tumor cells due to under-expression in tissues

List of RNAs interacting with ENST00000456917(MIR155HG)

Rank	Ensemble ID	Name	Position (hg19)	MINENERGY				SumEnergy
				MinEnergy	Binding site(query)	Binding site(target)	location	
74	ENST00000553119	AL589743.1	chr14:19650043-19718563	-30.8	480-530	28-78	ncRNA	-210.9
75	ENST00000397359	CDH24	chr14:23516272-23526747	-30.7	1207-1256	3455-3504	UTR3	-291.4
76	ENST00000346128	TJP1	chr15:29991572-30114706	-30.6	574-624	892-942	CDS	-2244.3
77	ENST00000557772	ESR2	chr14:64697637-64749703	-30.6	1228-1277	2911-2960	UTR3	-1269.7
78	ENST00000564619	AP000997.2	chr11:115509282-115517680	-30.6	1249-1309	2328-2388	ncRNA	-1986.2
79	ENST00000511828	RP11-766F14.2	chr4:100557687-100575805	-30.6	1226-1278	2818-2870	CDS	-2574.4
80	ENST00000374011	AHDC1	chr1:27860815-27930143	-30.5	374-421	2769-2816	CDS	-478.7

Supplementary Fig. S3. Table of Interaction prediction software analysis results showing LncRNA-SERB/ENST00000456917 may influence ERβ via targeting its 3′UTR

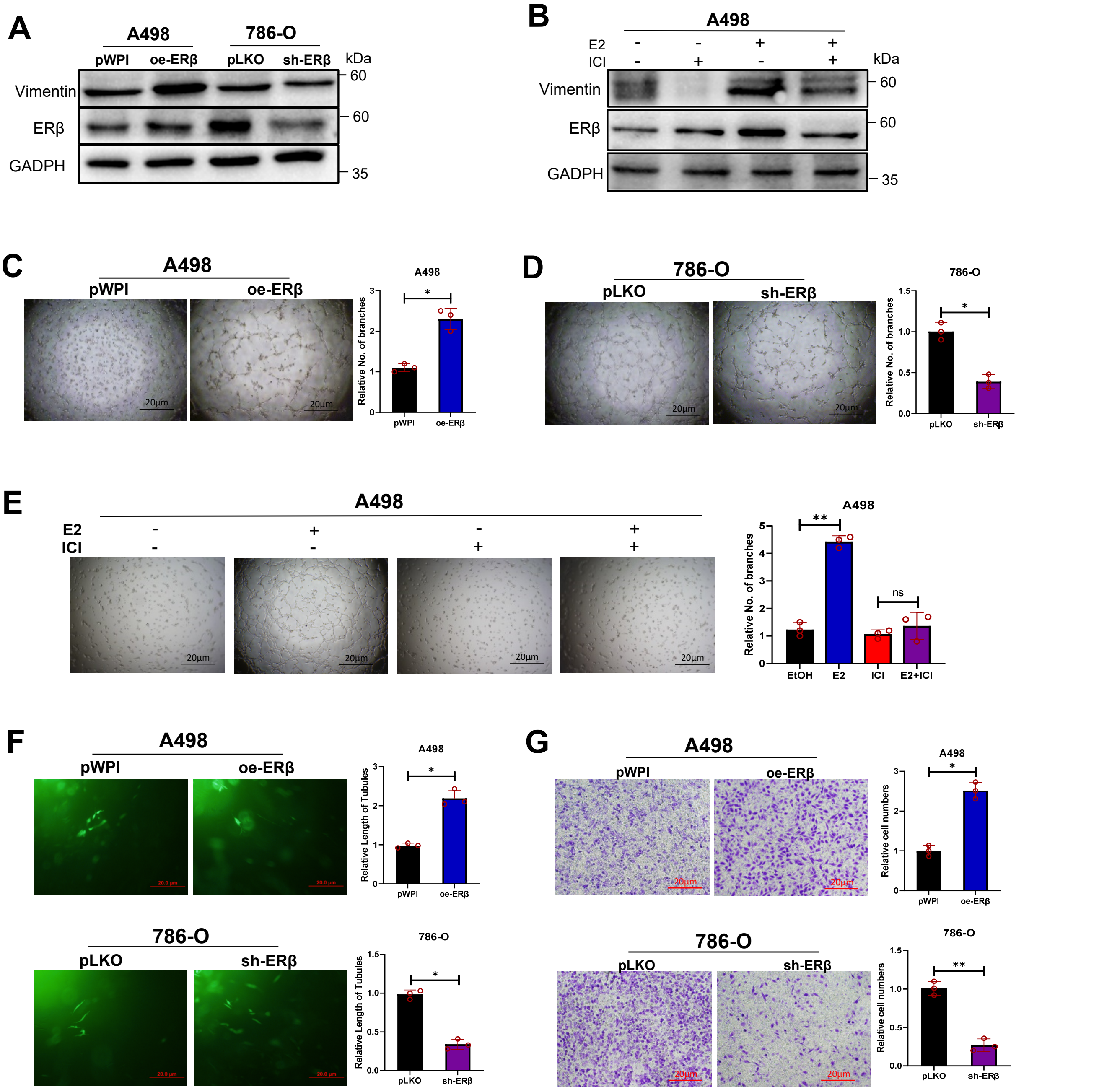

Supplementary Fig. S4. ERβ expression may influence VM formation and invasion in RCC

(A) Western blot validation of ERβ knockdown in 786-O cells and overexpression in A498 cells, and the expression level of Vimentin is positively correlated with ERβ. (B) Western blot verified that the ER agonist E2 and its inhibitor ICI 182,780 can also effectively affect the expression of ER and VIM. (C) 2D Matrigel-based tube formation assay proved that overexpression of ERβ in A498 cells can promote the formation of VM. (D) Knockdown ERβ in 786-O cells reduces VM formation in 2D Matrigel-based tube formation assay. (E) ERβ agonist E2 can increase VM formation, while its specific inhibitor ICI can reverse the former's effect on the formation of VM. (F) 3D Collagen I-induced tube formation assay results show that overexpress ERβ in A498 cells can promote cell length of tubules, while sh-ERβ in 786-O cells reduce cell length of tubules. (G) Invasion assay shows that ERβ leads to an increase in cell invasiveness.

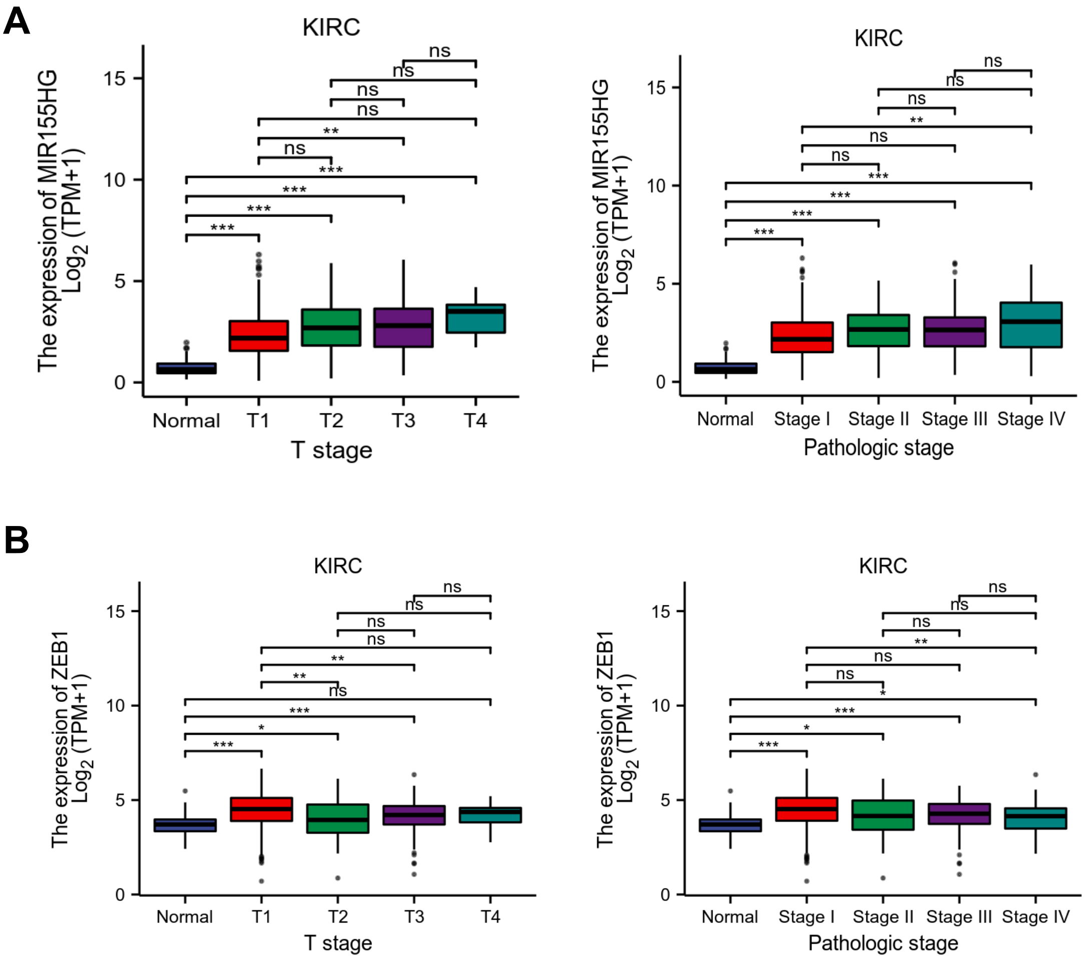

Supplementary Fig. S5. Results from TCGA database show that the expression of LncRNA-SERB and ZEB1 in renal cancer tissue is significantly higher than that in normal tissue

(A)The expression of LncRNA-SERB was higher in tumors than in normal tissues, although the difference between clinical stage and pathological grade was not statistically significant. (B) Expression of ZEB1 is higher in tumors tissues than in normal tissues also.

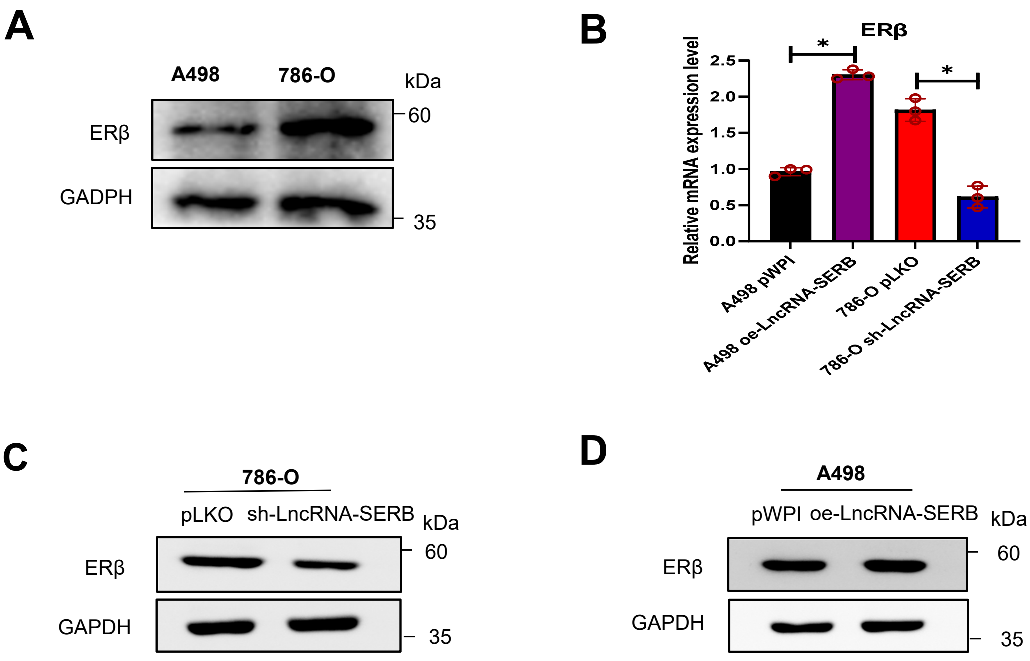

Supplementary Fig. S6. ERβ expression in A498 cells was relative lower than 786-O. Regulating LncRNA-SERB can alter ERβ expression in A498 and 786-O cells

(A)ERβ is relatively low in A498 cells but relatively high in 786-O cells.
(B)LncRNA-SERB can regulate ERβ expression in RNA level.
(C-D). LncRNA-SERB can regulate ERβ expression in protein level.

A

```
>10 dna:chromosome
chromosome:GRCh38:10:31315495:31318495:1CTCATTCCCCAGAGGGGTAAGAACCATTCTGTGGTAAACTATGTAACAAAAAT
CACATGGAAGTATGGAGAAAACATTTGTCCCTGTGATATCTCCTGGGAGGCTGTATTCGAAGGGCTGGCATGGGTCCTGTGGCCAACTGTGGG
TTCTGCATCACCCTGTAGTCATGTTTCCTTCCTGACTCAATGACTGCAGAATTTGGTGAGACTGCAACAGTGAAATGACCATTGCCCTCCTTT
GTTCCACGGTGAACTGCAAATCCAGAATTACTGTAGGTCAGGAGCCATGGTCAGAGACAAGAATTGAGTAGCGGCTTTATGGGACCTCTACAT
TCTGTACTTTGAGGGATAACATGTATCACATTCTTTTTCCTTCAAAAACTCTAAGATTAAAATTAGGTATTGAATTCAAGAGTTGCATTTACA
TGCTATCCTTCCATGAAATTAACGAACTTTAAATTTTGATGAAGACTAGAGAATTATACATTGCACTTCTCAATGTGTTTTGGAAGAGGTATG
TGGGAATGATATGCTTCATGTGGAACACCGTGAAATCTCTACATTTTAAACTTAAGATAATTTTATGTCAAATTTTATCCTAATTTATACAGG
AATCATATATGAAAAGCAAATAGGTGCAAAGGGTTTATGAAAAATTTATAGAAAAAACTCAGTATTGGAACTATTCTATCCATTTACATTTTT
CCCTGTAAATTTGGAATAGTATAGAAATTCATTTTTAGAATATCTTCCCTTCAGCATATATAATCCCAGTGCTCAGTAGAAGCAGTGTGTTAA
CATTTCATTGACATATTAAAAATAAGCATATATATTGAATGTATGCTGTATGACAGCACTACAATTACTAAAACCAAGAATGCAGGATACATG
TTAAAGTCATATATTAGTATCCCCTACCGTTTGATTTGAATAAACCTAAATTTTAATAATCTTCTTTTATAAATTGATTCTAAAGATTTTTCC
CCTATTCTTTAGCTGTATTTAAGAGATAAGAAGCAACCGTCACACATGCATCATGTTTAAAGCAGTAATTTATTCTGAGATTTACTTTTGTCT
GTTTTCTTTCCAGTCCAGGTATCAATATTTTCAAGAATATTAAAGTATAATAATCAAACATTCAATTCATAACATATATGCTTATCAGAAGCT
TTTACAAAATGGAAATTTTATGTTAGAAGATTTGGGCTACCAGTAATATTCACTGTGCATATTTTAAAAAAAAAGGTGAACAGAGTTCATTGT
TTAGGGAAAATGATTATGGAGAAACTAAAATCAAGAGCAATTTTAATGTGGATATTAATGACAGTGATCACTTTCATATCCATGACTTTATCT
GTTCCTAAGGACTAAAGGGATGAAGGCAGATTATGTACCTTACTGTCAAGAACAGGGTCAGAAAAGGTCAACAATATTAATTTAAGAAATGTT
TATTAAGCATCTACTATTGGCTACGTACTCCACTAGGTGTTGAGGACATAGAAATAAGAAAAAAAAGTTACCTGAAAAGGTACTTGTCCACAG
TTTGGCCCAAACTAACTAAACCAGAAACCACTAAAGTAAGGAAGAAAATGACTTCTGTTTGTCTCTTCACTTCATCTCTCTATGAGGTGAGGA
TGAACACTGAAGGAAGCTTAGCAGGGAGGTACAGGCCAGAAAGAGAACATGAGAAAATGCTATTTGTAATACCTCCTAAGTTTAAGAGTAGGT
AAAATGTGTGATAGAGCTGGAAAGTTTTTTTTTCCTTTCTGGTCAGAAATCAGGGTAGCTGTAAAAATGTTGGTGGGGTGGGGTCAATTCCAT
AGTCTATGACCTGATTCGGTAGGCGACTGAAAAACATTGTACAACTCTTAAAAAAAGGGAGGAAGAACTCACACTTAACCAAGCAAAGCAAGG
ACACCGTGTTGACATACACAAGTTTCGGTTAACATGGTATCAAGGAAAGCGAGGACTGCCTGTACTTAAAATGATCATTAAGACTAATGATTC
TAGTTTTGGCATTGAGGATGAATGCAGATATATAGACATAATGTATAGCAAAGAATATTTACAGTCACATTTCATGGACCAATAAATAACGCA
TTTACTTCTCAAAATGCCCAAGAAAATGTGGGAGAGAAAATGTTCATTTATATTCAAGATTAAACTTTCTAACTTTAAATCATCAGACTTCAG
CTGTAAATTTATTTCAGTTGAAAGGTTTGAAGACATTATTAATGAATATTCTAAAATTCCGGAAGGTGTTTAAAAGATAATTTTAACCTAATA
AAAAAATCCTGCCATAGAAGTGACAAAAATTATAAGCATAAATTTATTGAGCACCTACTATGTGTCATTGTGCTAGGTGATGTACTACGTTAT
TTCCAATACTCCGGTCACGTTTCAGTTTTCTCAATTTCTCTATCAATAACTGCTACATTGTTAATTTATAACTATATTACTTGAAATACAGCT
CAGCTTATTTATTCCATAAAGTTGGTCCCAAAGACGTTTCCTTATTCGAAGGAGGTGGGAAGCAGGAAGGAAACCTAAATCTAAATTCTACTT
AATACTGAAGACGACTTTCTTGATATTTGTGGTTATCTGTATGGTCTTTTCAGAAATCCCAAAACTTGTACCAAGTCAAGGATAAAATAAGAT
AAAATCAGCAATCTATCAGGTTCAGAGATCACATCTGTCAGCCGATGCTTCTTGCCTTAAGGTCCTGCACGGCGATGACCGCTCATTTAGGAA
GGAATTCATGGCCTGTGGATACCTTAGCTCTGAGTCCTGCCACCTAGGATCCCACGGTTCTACGCGAGGAAGAGGGCGGGGAGCGCGGACCGG
GTGTGGGAGGCCGAGGTGACAGCAGGTGAGGGCCGGGTGCGGATGGGGAAGTGAGACAAGCACCGTGTGGGTATTACTCATTCCGCTCTACTA
AGGAGGCTGCTGGCAAGCGGAACTTCTAGCCTCTCTTTCAATCCAGCTGAAGTTCAATCTCATTG
```

B

WT:
F:CTATCGATAGGTACCGAGCTGTAAGAACCATTCTGTGGTA
R:CCAAGCTTACTTAGATCGCACAATGAGATTGAACTTCAGC

C

MT:
F:GGGTCAGAAAAGGTCAACAATATTAATTT
GGATCC
R: GACCTTTTCTGACCCTGTTCTTGACAGTA

F:GGGTCAGGATCCGTCAACAATATTAATTT
R:GACGGATCCTGACCCTGTTCTTGACAGTA

Supplementary Fig. S7. Wild-type and mutant-type primers designed for estrogen-response-elements on the ZEB1 promoter sequence

(A)ZEB1 promoter sequence obtained from Ensembl websites.
(B)Wild-type of ERE#2 located on ZEB1 promoter sequence.
(C)The key sequence of ERE#2 was replaced by the BamH1 restriction site as a mutant-type.
